# From detection to action: ctDNA-MRD surveillance and translational strategies in early breast cancer

**DOI:** 10.3389/fonc.2026.1850732

**Published:** 2026-08-24

**Authors:** Jing Feng, Yujun Tong, Zhen Zhang, Ye Zhang

**Affiliations:** 1 Department of Breast Center, Mianyang Central Hospital, School of Medicine, University of Electronic Science and Technology of China, Mianyang, Sichuan, China; 2 Department of Neurosurgery, Mianyang Central Hospital, School of Medicine, University of Electronic Science and Technology of China, Mianyang, Sichuan, China

**Keywords:** circulating tumor DNA, early breast cancer, fragmentomics, methylation, minimal residual disease, trial design, whole-genome sequencing

## Abstract

Recurrence remains a major cause of mortality in early breast cancer (EBC), and conventional follow-up often identifies relapse only after clinically detectable disease has emerged. Circulating tumor DNA–based minimal residual disease (ctDNA-MRD) testing offers the possibility of detecting molecular relapse earlier and refining recurrence-risk assessment during follow-up. This narrative review examines the evolving role of ctDNA-MRD in EBC, focusing on assay interpretation, longitudinal surveillance, MRD-guided trial design, and clinical implementation. Prospective studies consistently show that postoperative or surveillance ctDNA positivity is associated with an increased risk of recurrence. However, test performance and interpretation vary with assay characteristics and sampling strategies, and whether treatment initiated solely on the basis of MRD positivity can improve patient outcomes remains unresolved. The central challenge is no longer simply to detect residual disease earlier, but to determine when and how that information should influence care. Further prospective validation, assay standardization, clear pathways for uncertain findings, and patient-centered implementation will be needed before ctDNA-MRD can be integrated into routine management of EBC.

## 1 Introduction

Outcomes for early breast cancer (EBC) have improved substantially with standardized surgery and (neo)adjuvant systemic therapy, yet the risk of mid- to long-term recurrence remains clinically meaningful (1). The latest overview from the Early Breast Cancer Trialists’ Collaborative Group (EBCTCG) indicates that patients with 1–3 positive lymph nodes have an approximate 10-year distant recurrence risk of 15%, while even node-negative patients retain a residual risk in the range of 7%–10% (2). Conventional follow-up relying on symptom-triggered imaging and serum biomarkers often detects relapse only after macroscopic disease becomes apparent.

Growing evidence from prospective studies and systematic reviews suggests that personalized circulating tumor DNA–based minimal residual disease (ctDNA-MRD) assessment can provide a “molecular warning” months to up to a year earlier than imaging. Importantly, the lead time is strongly dependent on both temporal window and tumor subtype: detection rates differ across the early postoperative period, the post-treatment phase, and long-term surveillance; they also vary among hormone receptor–positive (HR+), triple-negative breast cancer (TNBC), and human epidermal growth factor receptor 2–positive (HER2+) subtypes, and are jointly influenced by the assay platform and sampling schedule (3–5). In addition, sensitivity appears to be site-dependent. Cross-tumor studies of brain metastases indicate that, due to the blood–brain barrier and tumor-shedding characteristics, plasma ctDNA detection is less likely in central nervous system (CNS) disease, whereas cerebrospinal fluid (CSF) ctDNA can substantially improve detectability (6). However, routine CSF collection is impractical in breast cancer surveillance, and the current evidence base remains preliminary with limited sample sizes. In representative high-risk HR+ populations, conversion to ctDNA positivity is strongly associated with subsequent distant relapse, reinforcing the potential value of ctDNA as a prognostic “molecular sentinel” and as a biomarker for investigation in MRD-guided clinical trials (4, 5).

As of 2024–2025, both the analytical performance and the clinical deployability of ctDNA-MRD testing have improved in parallel. First, tissue-informed, personalized single-nucleotide variant (SNV) panels have achieved lead times measured in years in selected patients who later developed advanced relapse (7). Second, tissue-free methylation and multi-omic approaches have reported high specificity in population-based surveillance, can quantify lead time across prespecified time windows, and do not require tumor tissue (8). Third, whole-genome sequencing (WGS)–powered personalized tracking can interrogate thousands of patient-specific variants in a single workflow (median ~1,451 *loci*), enabling parts-per-million (ppm)–level detection with a median lead time of approximately 15 months; within the same cohort, this strategy has shown stronger clinical association than exome-based tissue-informed panels (9).

Despite these advances, improved detectability alone does not guarantee better outcomes. Preliminary findings from the phase III ZEST trial indicated that lower-than-anticipated ctDNA positivity and the frequent detection of metastatic disease at the first staging imaging assessment limited trial feasibility and contributed to early termination (10, 11). Together, these findings highlight that the proportion of patients identified as MRD-positive may be influenced by assay sensitivity, the sampling schedule, and risk enrichment of the study population. However, the available preliminary data do not allow the relative contributions of these factors to be clearly separated. These considerations, in turn, affect the feasibility of accrual and statistical power in MRD-intervention trials.

These technological advances, together with the unresolved challenge of translating molecular detection into clinical benefit, frame three interrelated questions addressed in this review. First, assay selection: we comprehensively compare the analytical characteristics and clinical use cases of tissue-informed, tissue-free, and WGS–powered ctDNA-MRD approaches (7–9). Second, surveillance strategy: to avoid simplistic cross-study comparisons, we adopt a unified framework that integrates the triad of threshold, depth, and sampling time point with longitudinal trajectories. Drawing on lessons from prospective studies such as c-TRAK-TN and LIBERATE, we discuss pragmatic workflows that combine an early postoperative baseline with repeated monitoring every 3–6 months, alongside short-interval confirmatory resampling followed by imaging when indicated (8, 12). Third, implementation and policy: using the NHS/NICE *ESR1* ctDNA pathway as an illustrative model, we examine key considerations for real-world adoption of MRD testing, including service delivery, reimbursement, and external quality assessment (EQA) and reporting standards, while also addressing challenges related to clonal hematopoiesis of indeterminate potential (CHIP), cost-effectiveness, and patient experience (8, 13).

## 2 Review methodology

This narrative review was informed by targeted searches of PubMed/MEDLINE and ClinicalTrials.gov for English-language publications from January 2012 to October 2025, supplemented by reference-list screening and selected official conference materials, regulatory guidance, consensus statements, and health-system documents. Search terms included “circulating tumor DNA,” “ctDNA,” “minimal residual disease,” “molecular residual disease,” “early breast cancer,” “surveillance,” “molecular relapse,” “methylation,” “fragmentomics,” “whole-genome sequencing,” “ctDNA clearance,” and “MRD-guided intervention.”

We prioritized prospective studies, randomized trials, longitudinal cohort studies, assay-comparison studies, systematic and methodological reviews, and consensus or regulatory documents addressing assay performance, recurrence prediction, surveillance strategies, trial design, or clinical implementation in EBC. Evidence from metastatic breast cancer and other solid tumors was included selectively when it provided relevant models for biomarker-guided treatment, endpoint selection, patient management, health-economic evaluation, or real-world implementation. Key studies were selected according to their methodological relevance, clinical relevance, applicability to the aims of this review, and recency where appropriate.

## 3 MRD assay landscape and key comparisons

Current ctDNA-MRD strategies in EBC can be broadly classified into three categories. First, tissue-informed personalized tracking uses the somatic mutation profile derived from surgical or biopsy tumor tissue as the patient-specific reference and longitudinally monitors customized *loci* in plasma. Second, tissue-free methylation and fragmentomics approaches do not require tumor tissue; instead, they infer MRD directly from aberrant methylation patterns and fragmentation features of plasma cell-free DNA (cfDNA). Third, WGS–powered ultra-sensitive approaches leverage genome-wide information to construct a high-density library of trackable features, enabling stable detection even at very low tumor fractions.

These three approaches differ substantially in pre-analytical handling, library preparation, thresholding and calling rules, management of CHIP, and reporting conventions. Accordingly, simplistic cross-study head-to-head comparisons are often misleading, and interpretation should consistently be anchored to the context defined by the triad of threshold, effective depth, and sampling time point (9, 14). Methodologically, the tissue-free branch is grounded in foundational work on cell-free methylated DNA immunoprecipitation and sequencing and the development of cfDNA fragmentomics (15–19). These assay pathways are summarized in **Table 1**.

**Table 1:** Overview of three ctDNA-MRD assay pathways.

Assay pathway	Tissue require	Core signal	Indicative LoD (contextual)	Indicative lead time (contextual)	Specificity / PPV (contextual)	Turnaround & deployability	Typical use cases
Tissue-informed	Yes	Patient-specific SNV/indel tracking (multi- locus); UMI error suppression + statistical model	≈1x10^-5^ ; depends on loci, unique depth, error model; not cross-study comparable	Months to ≥12 mo; depends on subtype and sampling cadence	High; confirm borderline positives (re- draw 2-4 wk ± imaging)	Moderate; mid- high cost; bespoke workflow; best centralized	High-risk surveillance; platform trials; tissue available; maximize sensitivity
Tissue-free	No	Methylation + fragmentomics (size/end bias/nucleosome ) with ML inference	Algorithm- dependent; driven by thresholds + sample QC; suited to low shedding/no tissue	Typically months (median ~5 mo in some analyses)	Generally high in prospective cohorts; interpret within prespecified time windows	Fast; moderate cost; standardized library + cloud analytics; scalable	Routine outpatient surveillance; tissue unavailable; rapid rollout
WGS-powered	Yes	Large private SNV set (~10^3 loci); ppm-level quantification; optional duplex	ppm-level; driven by loci, coverage, error model	Longer (median ~15 mo in-cohort; vs exome- informed panels)	High; stronger association with RFS-like endpoints (within cohort)	Slow; high cost; centralized; strict EQA	High-risk cohorts; adaptive platform trials; need longest lead time/lowest LoDH

LoD, limit of detection; PPV, positive predictive value; SNV, single nucleotide variant; UMI, unique molecular identifier; RFS, relapse-free survival; EQA, external quality assessment; ppm, parts per million (per haploid genome equivalents, hGE). Contextual metrics vary by platform, locus count, unique-molecule/coverage depth, error model, and sampling schedule, and tumor subtype; direct cross-study comparisons are inappropriate. Positives should be re-sampled in 2–4 weeks with imaging as indicated.

### 3.1 Tissue-informed tracking

This strategy typically begins with whole-exome sequencing (WES) or targeted panel sequencing of surgical or biopsy tumor tissue to identify patient-specific SNVs and insertions/deletions (indels). Longitudinal plasma samples are then profiled using deep sequencing—most commonly multiplex PCR or hybrid-capture approaches incorporating unique molecular identifiers (UMIs) for error correction—followed by variant calling based on an error model and prespecified positivity thresholds. A key strength of this approach is that ultra-deep interrogation of known mutations can markedly suppress background noise and yield high within-patient consistency. Its limitations include reliance on adequate, high-quality tumor tissue; customized assay design with longer turnaround time and higher cost; and the risk of false-negative results in the setting of limited tissue, pronounced intratumoral heterogeneity, or extended surveillance, where target *loci* may be missed due to locus dropout or clonal evolution (14).

The prospective c-TRAK-TN study demonstrated the feasibility of longitudinal monitoring on a quarterly (every 3 months) schedule and suggested that earlier sampling and more frequent blood draws can reduce cases in which the first ctDNA-positive result coincides with overt metastatic disease, thereby increasing the proportion of patients with actionable positivity (12). In parallel, cancer personalized profiling by deep sequencing and its integrated digital error suppression (iDES) framework have shown high sensitivity across multiple solid tumors and provide important methodological foundations for SNV-panel–based MRD assays (20).

This approach is most applicable when high-quality tumor tissue is available, maximizing within-patient sensitivity is a priority in high-risk individuals, and workflows can be tightly controlled in single-center or platform-style trials (12, 14). It may be less suitable when tumor tissue is limited, rapid deployment is required, surveillance is prolonged in the setting of substantial heterogeneity, or turnaround time and cost constraints are substantial (14).

### 3.2 Tissue-free methylation and fragmentomics

Tissue-free approaches integrate aberrant methylation signatures and fragmentomic features of plasma cfDNA—including fragment-length distributions, end-motif or breakpoint biases, and nucleosome positioning—to infer MRD without requiring tumor tissue. This enables a streamlined surveillance workflow from blood draw to library preparation and algorithmic interpretation. In longitudinal monitoring of EBC, prospective studies have reported high specificity and quantifiable lead time across prespecified time windows. In particular, methylation-dominant MRD has outperformed genotype-only MRD based on fixed mutation panels, and combining the two has not yielded a clear incremental benefit (8). Moreover, prospective trials such as LIBERATE have suggested strong short-term predictive performance, with a positive predictive value (PPV) of 100% (21). Foundational studies further support the biological rationale for this strategy: cfDNA nucleosome footprints can reflect tissue of origin (18), and fragmentation patterns can materially improve detection sensitivity (17, 19).

Methodological reviews have emphasized that tissue-free assays may be especially advantageous in settings characterized by low tumor shedding or unavailable tissue, but the clinical utility of intervention triggered solely by MRD positivity still requires confirmation in randomized controlled trials (3, 22).

These assays may be particularly useful when tumor tissue is unavailable or impractical to obtain, when deployment is intended for large-scale prospective surveillance cohorts, or when shorter turnaround time and a standardized workflow are priorities (8, 22). Borderline positive findings should prompt short-interval repeat sampling, with imaging when clinically indicated. Reports should transparently disclose algorithmic thresholds, sequencing depth and/or unique-molecule depth, and sample pass rates. Although tissue-free approaches may be less susceptible to CHIP-related interference, methods should still specify how hematopoietic-derived signals are identified and mitigated (8, 22, 23).

### 3.3 WGS–powered approaches

WGS–powered strategies typically use baseline WGS to identify thousands of patient-specific variants in a single step (median ~1,451 *loci*, with an upper range of ~1,800 *loci* per individual). Subsequent surveillance samples undergo ultra-deep longitudinal profiling, and signal is recovered through error suppression and statistical aggregation to achieve ppm–level detection. Here, ppm denotes the number of mutant molecules per million haploid genome equivalents (hGE). Error modeling and duplex sequencing can further improve the detection of ultra-low-frequency variants, providing methodological support for ppm-scale analyses (24).

Available clinical evidence includes a prospective breast cancer cohort in which quantitative ctDNA levels spanned approximately 2.19–204,900 ppm, with 39% of samples below 100 ppm. The median lead time to clinical relapse was approximately 15 months, MRD positivity was significantly associated with relapse-free survival (RFS), and the WGS–powered approach outperformed an exome-based tissue-informed panel in terms of both detection rate and lead time within the same cohort (9).

Complementary WGS applications include low-pass and ultra-low-pass WGS (LP-/ULP-WGS), which can be used to estimate tumor DNA fraction and track copy-number alterations (CNAs) using tools such as ichorCNA (25, 26).

Implementation of WGS–powered MRD testing is resource-intensive, with substantial requirements for library preparation, computational capacity, and data storage. WGS–powered approaches may therefore be most appropriate for high-risk cohorts and platform-style adaptive trials in which maximizing lead time and lowering the detection limit are primary objectives, ideally accompanied by standardized reporting and health-economic evaluation (9, 14, 24). They may be less suitable in resource-constrained settings or when rapid turnaround is required. Trial protocols and reports should prespecify and disclose key parameters, including the number of tracked *loci*, effective coverage or unique-molecule depth, sample-level scoring metrics and confidence, and the limit of detection/limit of quantification (LoD/LoQ) for negative results (9, 14, 23).

### 3.4 Shared principles across MRD assays

Integrating evidence from recent prospective studies, expert consensus statements, and EQA experience, several shared principles should be applied across different MRD assays. The positivity threshold is jointly influenced by the number of tracked *loci*, unique-molecule depth and/or effective coverage, and the underlying error-suppression model. Reports should present the triad of threshold, depth, and sampling time point side by side, and clinical interpretation should be anchored in longitudinal trajectories rather than a single time point, to avoid misleading cross-study comparisons (3, 14, 20, 23, 24). For monitoring strategies based on LP-/ULP-WGS or WES, the approach used to estimate tumor fraction and large-scale somatic CNAs, together with the relevant detection limits, should also be specified (25, 26).

After a first conversion to ctDNA positivity, repeat sampling within 2–4 weeks has been proposed as a confirmation strategy in prospective studies, coupled with imaging when clinically indicated. Protocols should prespecify repeat-positivity rules (e.g., recurrence at ≥ 2 *loci* or a composite score exceeding a predefined threshold) and the downstream management pathway. Earlier baseline sampling and more frequent longitudinal sampling can increase the proportion of patients with actionable MRD positivity. This workflow is supported by prospective data from c-TRAK-TN and by time-windowed reporting from tissue-free studies (8, 12, 14, 21).

For SNV-dominant assays, paired leukocyte sequencing or filtering against a clonal hematopoiesis gene set—commonly including *DNMT3A, TET2, ASXL1, PPM1D*, and *TP53*—should be implemented, with explicit documentation of blacklist rules, the error model, and exclusion thresholds in both methods and reporting. Although methylation and fragmentomics approaches are theoretically less susceptible to interference from CHIP, they should still describe how hematopoietic-derived signals are identified and mitigated, and whether such controls were applied in reporting. These requirements are explicitly recommended in the ELBS reporting consensus and methodological reviews (14, 22, 23).

Routine participation in EQA/proficiency testing (PT) is recommended, covering the full workflow from pre-analytics and library preparation to bioinformatic calling and reporting. Prospective protocols should prespecify the longitudinal sampling schedule, confirmation procedures, thresholds, and primary endpoints—prioritizing distant recurrence and lead time—to ensure end-to-end alignment between assay performance and clinical utility trials. Multi-center EQA evidence indicates that this approach improves cross-site comparability and reproducibility (14, 23, 27). Minimum reporting and quality requirements are outlined in **Table 2**.

**Table 2:** Minimum reporting and quality requirements for ctDNA-MRD assays.

Module	Minimum items (key points)	Reporting format / acceptance criteria
Sampling & timepoints	Define post-operative baseline and end-of-(neo)adjuvant baseline windows; longitudinal cadence; imaging timing; sample handling and TAT; confirmatory repeat draw.	Dates (YYYY-MM-DD); cadence q3-6 mo; imaging same day or ±7 d; repeat draw for positives at 2-4 wk.
Thresholds & interpretation rules	Prespecify positivity definition and decision rule (score/locus recurrence); borderline band and handling; rules for 'tumour-uninformative' cases (if applicable).	Numerical threshold with statistical basis; report handling when no tumour variants detected; state that cross-study/platform comparability is inappropriate.
Coverage & UMI depth	Report mean/median coverage and UMI depth per sample; minimum pass cut-off; QC failure flagging and failure rate.	Mean/median coverage (×) and UMI counts; minimum pass cut-off (e.g., ≥8x10^4^ UMIs); flag QC failures.
LoD / LoQ	Define LoD/LoQ and estimation method; applicability to MRD calling; false-positive control and dynamic range (as relevant).	LoD and/or LoQ (% or VAF); specify dilution/simulation versus statistical modelling; report assumptions.
Tracked loci & effective coverage (personalised/WGS)	Total variants/loci tracked per patient; effective coverage; rule for calling MRD (e.g., recurrent loci requirement).	Median tracked loci and effective coverage; prespecified positivity logic in the SAP (e.g., ≥2 recurrent loci).
Algorithm thresholds & pass rate (tissue-free)	Classifier/score definition (methylation/CNV/etc.); thresholds; pass rate; borderline handling and confirmation pathway.	Thresholds and cohort definition; report pass rate; repeat draw ± imaging for borderline positives.
CHIP control	WBC-paired sequencing and/or blacklist strategy; handling of CH-associated variants; residual risk statement.	Describe CHIP control method; state whether applied; quantify filtered calls if available; note residual risk.
Error suppression / modelling	UMI/duplex strategy; background error model; inter-/intra-batch reproducibility; key sources of noise.	Report error rate (~10^-n^) and model/version; inter-/intra-batch reproducibility metrics.
Units & quantitation range	Reporting unit (VAF, hGE/mL, molecules/mL, etc.) and quantitation range; conversion rules; lower cut-off handling.	Unit and lower limit; demonstrate consistency; describe conversions and handling below cut-off.
Quality control (QC)	Plasma volume / cfDNA input; haemolysis/contamination checks; library/sequencing QC; failure handling.	QC thresholds; minimum input; sample failure rate; handling and reporting of failed/invalid samples.
Repeat positives & confirmation pathway	Repeat draw window and criteria for 'confirmed positive'; imaging indications; pathway aligned with SAP.	Repeat draw at 2-4 wk; imaging as indicated; prespecified pathway aligned with the SAP.
Software / versions / parameters	Pipeline/software name; version; key parameters; code availability and auditability.	Software and version; parameter list; code/DOI/repository or auditable closed-source route.
EQA/PT participation & results	External quality assessment/proficiency testing participation status; most recent outcome; corrective actions.	Programme and round; pass/fail; corrective actions; date of last participation.
Data traceability & archiving	Raw/intermediate/final files; report version and timestamp; retention and access control.	Accession or path for fastq/bam/vef; report version and date; retention period and access control.

Items reflect minimum disclosure; align prospectively with the SAP and operational workflow. Present LoD/LoQ and positivity thresholds alongside coverage/UMI depth and sampling timepoints. Cross-study/platform comparisons are inappropriate.

CHIP, clonal haematopoiesis of indeterminate potential; CI, confidence interval; cfDNA, cell-free DNA; d, days; EQA, external quality assessment; hGE, haploid genome equivalents; ICC, intraclass correlation coefficient; LoD, limit of detection; LoQ, limit of quantification; mo, months; ppm, parts per million (per hGE); QC, quality control; SAP, statistical analysis plan; TAT, turnaround time; UMI, unique molecular identifier; wk, weeks; WBC, white blood cell; WGS, whole-genome sequencing; VAF, variant allele fraction.

## 4 Surveillance schedule and key time points

In prospective studies, ctDNA-MRD surveillance has commonly incorporated an early postoperative and/or post-treatment baseline followed by longitudinal testing at intervals of approximately 3–6 months. Gains in both the proportion of MRD-positive cases identified and the molecular lead time are jointly determined by assay sensitivity and the testing interval (3, 28). A first post-operative blood draw at 2–6 weeks is commonly used to establish a negative baseline. In a 2024 multicenter prospective study, none of the landmark samples collected within 8 weeks after surgery were ctDNA-positive (0/46), suggesting limited sensitivity when sampling is too early; nevertheless, this baseline can be valuable for interpreting subsequent trajectories and calibrating background noise (28). A second baseline obtained 2–4 weeks after completion of (neo)adjuvant therapy can capture treatment-related clearance kinetics and provides a consistent reference for later comparisons (5).

Subtype-specific recurrence patterns suggest that a uniform testing schedule may not be appropriate. In the same 2024 cohort, the median interval from surgery to the first molecular signal of relapse differed markedly across subtypes—approximately 25 months for HR+, 17 months for HER2+, and 6 months for TNBC. In contrast, for cases with brain metastases, the molecular-to-clinical lead time was only 3.8–5.6 months, underscoring both site and subtype dependence (28). These observations provide a rationale for evaluating quarterly monitoring in TNBC and HER2-positive disease and testing every 3–4 months during the first two years, followed by less frequent monitoring, in HR-positive disease. However, these subtype-specific intervals have not been prospectively established as optimal surveillance schedules. A separate 2024 TNBC study provides additional rationale for evaluating intensified surveillance during the early post-treatment period to improve the timely detection of molecular relapse (29).

Assay capability also shapes the optimal monitoring interval. In breast cancer surveillance, tissue-free methylation and fragmentomics assays have reported specificity and positive predictive value of 100%, with a median lead time of roughly 5 months presented across prespecified time windows, making them attractive for evaluation in longitudinal surveillance studies and future outpatient implementation programs—provided that positive findings are confirmed with short-interval repeat sampling (8). For high-risk populations where maximizing lead time and pushing the lower limit of detection are priorities, WGS–powered personalized tracking can monitor ~1,451–1,800 *loci* per patient, quantify ctDNA across a range of ~2.19–204,900 ppm, and has reported a median lead time of ~15 months with a significant association between MRD positivity and RFS. These features provide a rationale for evaluating earlier baseline sampling and quarterly testing in platform-style trials and high-risk prospective surveillance cohorts (9).

In prospective surveillance protocols, an initial conversion to ctDNA positivity may be followed by short-interval repeat sampling within 2–4 weeks, with imaging when clinically indicated, to confirm the molecular signal before an intervention trigger is activated. Increasing plasma input or incorporating technical replicate testing may further improve the detection of low-abundance signals in research settings (8, 30, 31).

**Figure 1:**
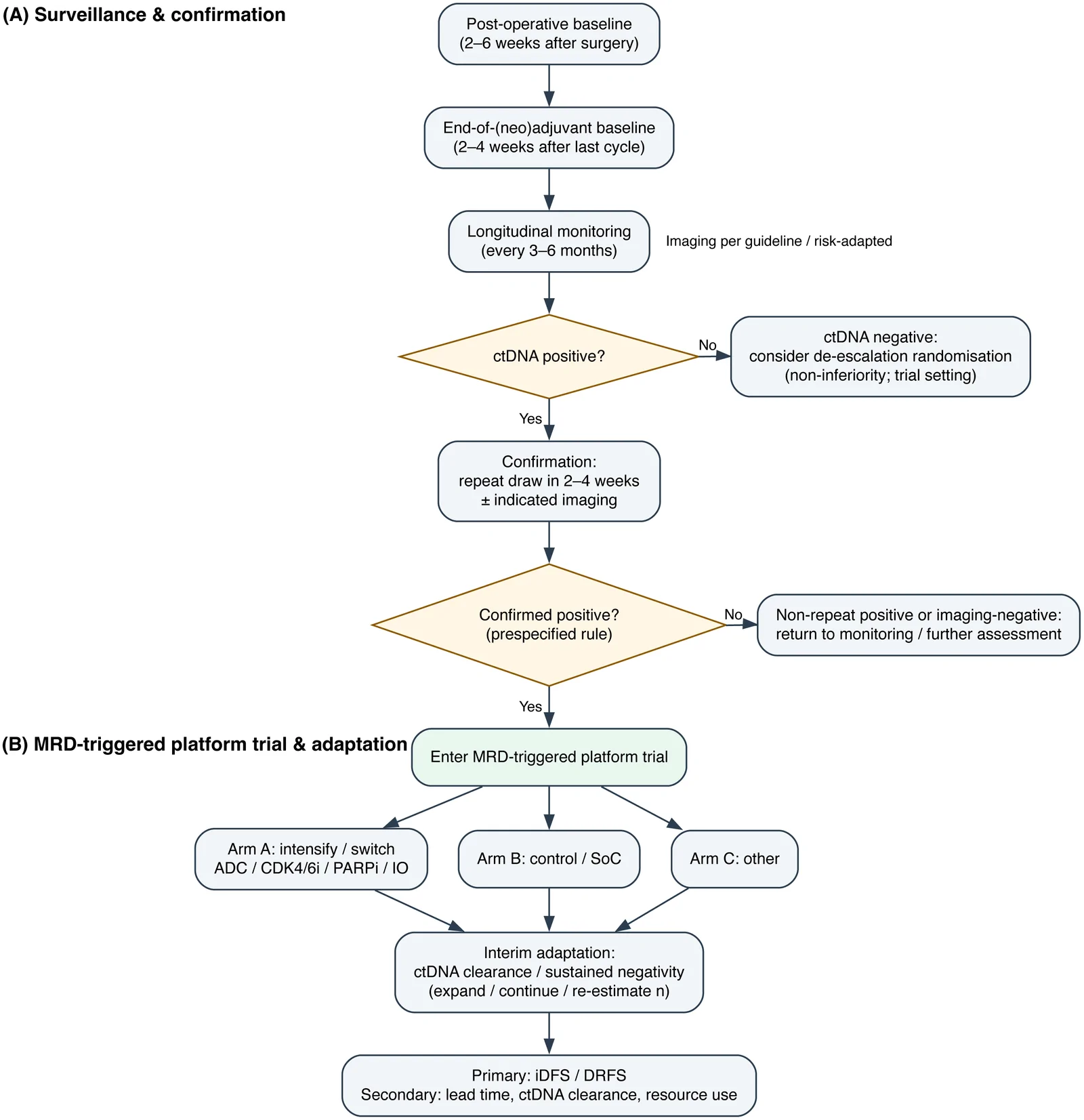
Proposed framework for ctDNA-MRD surveillance, confirmation, and MRD-triggered clinical trials in early breast cancer. **(A)** Surveillance and confirmation include postoperative and end-of-(neo)adjuvant baseline assessment, longitudinal ctDNA monitoring, and short-interval repeat sampling with imaging when clinically indicated after an initial positive result. **(B)** Confirmed MRD positivity may serve as an eligibility criterion for prospective MRD-triggered platform trials, with treatment escalation or switching evaluated against standard-of-care control strategies. ctDNA clearance or sustained negativity may be explored for interim adaptation, while iDFS and DRFS remain key clinical endpoints. The proposed framework is intended for prospective trial evaluation and does not represent an established clinical management pathway.

Taken together, available evidence provides a basis for prospectively evaluating postoperative and end-of-treatment baseline sampling followed by subtype- and assay-informed testing at intervals of approximately 3–6 months. In prospective studies, an initial ctDNA-positive result may be followed by short-interval confirmatory sampling and imaging when clinically indicated. The optimal testing intervals, confirmation criteria, and duration of surveillance remain to be established through prospective validation. For prospective trials, the testing schedule, confirmation rules, and resource requirements should be prespecified in the statistical analysis plan to ensure feasibility and clinical interpretability. A proposed surveillance and decision framework is illustrated in **Figure 1**.

## 5 From detection to decision

### 5.1 Molecular detection and clinical actionability

A central lesson from ZEST, the first phase III MRD-intervention trial, is that trial feasibility is highly contingent on the observed ctDNA-positivity rate and the monitoring schedule. Among 2,746 patients prescreened, only 8% (147/1,901) were ctDNA-positive, and 49% (72/147) already had metastatic disease detected at the first staging imaging assessment. This combination of low positivity and a high proportion of imaging-positive cases at initial staging contributed to early trial termination (10, 11).

The low ctDNA-positivity rate may have reflected several interacting factors, including the analytical sensitivity of the assay, the timing and frequency of blood collection, and the clinical characteristics and recurrence risk of the screened population. However, the available preliminary reports do not permit these contributions to be reliably distinguished, and none should therefore be considered the sole explanation for the observed positivity rate. Methodological limitations related to assay sensitivity or sampling schedule could reduce the detection of low-abundance or transient ctDNA signals, delay identification of molecular relapse, and increase the likelihood that metastatic disease is already radiographically evident when ctDNA positivity is first confirmed. Conversely, insufficient enrichment for patients at higher risk of recurrence could lower the overall prevalence of detectable MRD and substantially increase the number of patients required for screening and randomization.

These findings support prospective evaluation of more sensitive assay strategies, earlier and longitudinal sampling, and appropriately defined risk-enrichment criteria in future trials, while recognizing that their relative effects on MRD detection remain uncertain (10, 11, 32).

Even if these methodological refinements improve MRD detection, molecular detection should not be equated with clinical actionability. Although postoperative or surveillance ctDNA positivity is consistently associated with an increased risk of recurrence, it remains uncertain whether initiating or changing treatment solely on the basis of an MRD-positive result improves clinically meaningful outcomes in EBC. Therefore, the strategies discussed below should be interpreted as principles for prospective trial design rather than established management pathways.

### 5.2 MRD-guided intervention and longitudinal ctDNA dynamics

Translating improved molecular detection into clinical benefit will require prospective trials to determine whether intervention triggered by confirmed ctDNA positivity can improve patient outcomes. First, future trials should evaluate whether more sensitive assay strategies, earlier postoperative and post-treatment sampling, longitudinal testing, and enrichment for higher-risk populations can increase the proportion of ctDNA-positive patients identified before radiographically evident relapse. Second, endpoints should be aligned with the intervention hypothesis: iDFS and/or distant recurrence–free survival (DRFS) as primary outcomes, with ctDNA clearance or sustained ctDNA negativity prespecified as secondary endpoints or candidate early efficacy measures requiring prospective validation. Third, power and sample size should be prospectively parameterized based on the anticipated positivity rate, and the workflow should embed confirmatory steps—short-interval repeat sampling combined with imaging when clinically indicated—to reduce false triggers and avoid overtreatment (30).

PADA-1 provides an informative operational example of biomarker-triggered treatment switching. Using longitudinal ctDNA monitoring of estrogen receptor 1 (*ESR1*) alterations, patients were switched early from an aromatase inhibitor (AI) plus a cyclin-dependent kinase 4/6 inhibitor (CDK4/6 inhibitor) to fulvestrant plus a CDK4/6 inhibitor upon detection of an *ESR1* mutation, resulting in a significant improvement in progression-free survival (PFS). However, PADA-1 should not be regarded as direct evidence for postoperative MRD-guided intervention in EBC, because it evaluated emerging endocrine resistance in patients with established metastatic disease and a defined therapeutic target. Its relevance to MRD-guided trials is therefore primarily methodological, including biomarker confirmation, trigger definition, and trial workflow (33, 34).

Evidence linking ctDNA clearance to clinical outcomes has emerged from recent prospective and retrospective breast cancer studies emphasizing longitudinal dynamics. In TBCRC030, a study in early TNBC, failure to clear ctDNA by week 12 was significantly associated with higher residual cancer burden and an increased risk of subsequent recurrence, suggesting that ctDNA clearance may serve as a candidate early marker of treatment activity and could be evaluated as an eligibility or stratification factor in prospective escalation trials (35). A 2024 long-term follow-up study reported markedly better outcomes among patients who remained persistently ctDNA-negative, supporting the prognostic value of sustained negativity and its evaluation in ctDNA-guided de-escalation trials; importantly, the study also demonstrated the operational feasibility of semiannual blood draws over a 12-year surveillance period (5). In addition, the final analysis of IMpassion031 linked ctDNA dynamics to event-free survival (EFS), showing that perioperative immunotherapy increased ctDNA negativity rates and that postoperative ctDNA positivity identified the worst-prognosis subgroup among patients without pathologic complete response (non-pCR), providing empirical support for ctDNA clearance as a candidate proximal indicator of treatment activity (36). Consistent with these findings, ctDNA clearance and sustained ctDNA negativity could be prespecified as exploratory or secondary efficacy measures in prospective trials, although their validity as intermediate or surrogate endpoints remains to be established (14, 37).

Assay choice and the longitudinal testing interval also reshape the effective denominator of patients who can be triggered for intervention. In c-TRAK-TN–linked analyses and methodological comparisons, multi-locus personalized sequencing detected first ctDNA conversion earlier than simplified strategies, increasing the proportion of patients who remain eligible for MRD-directed intervention. This, however, requires protocol-level preregistration of repeat-positivity rules (e.g., recurrence at ≥ 2 *loci* or a composite score exceeding a prespecified threshold) and an explicit trigger pathway (repeat sampling followed by targeted imaging and then randomization) (38). A 2024 study further indicates that ctDNA positivity at even a single postoperative time point is significantly associated with distant metastasis–free survival, underscoring the contribution of early-window sampling to actionable positivity (39).

With respect to technical strategies that can increase the actionable ctDNA-positivity rate, tissue-free (methylation/fragmentomics) and WGS–powered approaches offer complementary advantages at opposite ends of the spectrum—deployability versus maximal lead time. In LIBERATE, the tissue-free strategy demonstrated high specificity and a clearly time-windowed lead time profile, making it well suited for integration into routine surveillance pathways; however, any positive result should be confirmed with short-interval repeat sampling to guard against transient fluctuations (8). By contrast, a 2025 prospective breast cancer cohort showed ppm-level detection with a median lead time of approximately 15 months using a WGS-powered personalized tracking approach, which outperformed an exome-based panel within the same cohort. These features support earlier baselining and quarterly monitoring in high-risk follow-up and platform-style escalation trials (9).

### 5.3 ctDNA-guided de-escalation strategies

The DYNAMIC prospective randomized trial in colon cancer provides proof of principle for ctDNA-guided de-escalation: postoperative management informed by ctDNA significantly reduced chemotherapy use while remaining non-inferior to standard management for recurrence-related outcomes, and the 5-year follow-up further reinforced long-term safety and efficacy under a non-inferiority framework (40, 41). However, DYNAMIC should be regarded as a trial-design precedent rather than direct evidence for ctDNA-guided de-escalation in EBC, given differences in disease biology, recurrence patterns, treatment duration, and the clinical consequences of false-negative MRD results. By extension, ctDNA-negative–guided de-escalation in breast cancer would need to be evaluated within a testable non-inferiority design. Whether sustained ctDNA negativity can justify reducing chemotherapy or targeted therapy intensity, or shortening the duration of endocrine therapy, must be evaluated in prospective randomized trials with iDFS and/or overall survival (OS) as primary endpoints, while explicitly quantifying false-negative risk, patient anxiety, and resource utilization. The 2024 FDA guidance similarly states that, in solid tumors, MRD is not yet an established surrogate endpoint; it may be used as a supportive measure for interim adaptation and pharmacodynamic assessment, but requires transparent reporting of the LoD/LoQ, repeatability, and predefined rescue pathways (42). Ongoing de-escalation or treatment-reallocation studies—such as ctDNA-guided chemotherapy de-escalation and endocrine optimization programs in HR-positive disease—offer practical templates and parameterized assumptions for trial design in this area (43, 44). In parallel, the 2024 Friends of Cancer Research white paper proposes a validation framework for using ctDNA dynamics as a potential intermediate endpoint in clinical trials, which may inform endpoint planning for MRD-focused studies (45).

### 5.4 Patient-centered considerations

Patient-centered safeguards should be incorporated before surveillance begins. Pre-test counselling and informed consent should explain the purpose and limitations of ctDNA-MRD testing, including the possibility of false-negative, borderline, or molecular-positive/imaging-negative results, as well as how and when results will be communicated (23, 46, 47). Patients should understand that earlier molecular detection does not necessarily indicate that an intervention with proven clinical benefit is available, and that treatment initiated solely on the basis of MRD positivity remains investigational in EBC (30, 42). They should also be informed that serial monitoring may involve additional blood draws, repeat imaging, and periods of uncertainty while confirmatory investigations are completed (46).

Indeterminate or equivocal findings should follow a prespecified pathway rather than trigger immediate treatment escalation. This pathway may include laboratory review of assay quality and possible CHIP-related interference, followed by repeated or orthogonal testing and, where appropriate, repeat blood sampling or imaging. Reports should distinguish an equivocal signal from confirmed MRD positivity, describe the uncertainty of the finding, and identify the clinician or multidisciplinary team responsible for interpretation and follow-up (23, 30). A clear timeframe for communicating results and arranging reassessment may help patients manage anxiety while awaiting confirmation (46).

Repeated monitoring may impose psychological and practical burdens. Waiting for results, receiving discordant molecular and imaging findings, and uncertainty regarding the next clinical step may increase anxiety, although some patients may also experience reassurance from closer surveillance (46, 47). Prospective studies should therefore assess patient understanding, anxiety, acceptability, and practical burden as patient-reported outcomes and should provide structured result communication and access to psychosocial support. Economic evaluations should consider the costs of serial assays, confirmatory testing, triggered imaging, and downstream care, while future implementation studies should also capture patient-level expenses and time commitments that are not adequately represented in current payer-based economic models (48–50).

### 5.5 Trial-design considerations

The following points represent proposed design principles for future MRD-guided clinical trials. A unified surveillance and confirmation framework could be used to evaluate biomarker- and subtype-informed intervention strategies. MRD-positive patients could be enrolled in matched escalation arms, whereas MRD-negative patients could be evaluated in separate randomized de-escalation cohorts with prespecified safety boundaries. iDFS and/or DRFS should serve as primary endpoints, while ctDNA clearance or sustained ctDNA negativity could be prespecified as exploratory or secondary efficacy measures (30, 42). Patient-reported outcomes, including anxiety and acceptability, should also be incorporated as secondary endpoints (46, 47). Core methodological elements—including prespecified positivity and confirmation rules, CHIP management, LoD/LoQ, and EQA/PT participation—should be embedded in the protocol and reported transparently (23, 27, 42).

For the q3 versus q6 monitoring comparison, we used a simple deterministic model (**Supplementary Tables 1**, **2**) that combines assay sensitivity, MRD prevalence, sampling delay, imaging triggers and unit costs to estimate tests per person-year, capture probability, triggered imaging, and incremental cost per additional MRD-positive case.

## 6 Implementation and policy: from *ESR1* ctDNA to MRD

Liquid-biopsy applications using plasma *ESR1* alterations to guide endocrine therapy switching have established a clear clinical precedent in metastatic breast cancer. The phase III EMERALD trial demonstrated improved PFS in patients with *ESR1*-mutant disease and linked plasma ctDNA results to treatment selection, supporting biomarker-guided endocrine therapy in this setting (51–53). In England, the NICE/NHS Genomic Medicine Service—delivered through the Genomic Laboratory Hub (GLH) network and the National Genomic Test Directory (NGTD)—has incorporated *ESR1* ctDNA testing into routine care. Since January 2025, testing has been routed through designated GLHs under test code M3.13, creating a formal access pathway for a plasma-first *ESR1* strategy (54–56). The relevance of this pathway to EBC lies mainly in its operational features rather than in direct evidence for MRD-guided treatment. In metastatic disease, *ESR1* testing identifies an acquired resistance alteration with an established therapeutic implication, whereas postoperative MRD positivity may precede radiographic relapse and currently lacks a validated standard intervention. Nevertheless, the *ESR1* pathway provides useful lessons regarding sample routing, assay standardization, reporting, turnaround time, and the integration of molecular results into predefined clinical workflows. The 2025 European Liquid Biopsy Society (ELBS) consensus further proposes a minimum reporting dataset, that includes calling thresholds, unique-molecule depth and/or sequencing depth, sampling time points, the LoD/LoQ, management of CHIP, and software/version traceability. It also recommends short-interval repeat sampling with imaging when clinically indicated for equivocal results (23). Evidence from EQA studies indicates that workflow standardization and proficiency verification can improve inter-laboratory consistency (27). Together, these principles may inform the design of future MRD surveillance programs, although their clinical utility in EBC remains to be prospectively established.

Translating these operational lessons to EBC would require a predefined end-to-end pathway linking test eligibility, sample routing, laboratory processing, reporting, multidisciplinary review, and clinical follow-up. Systematic reviews of liquid-biopsy implementation identify laboratory infrastructure, sample routing, turnaround time, standardized reporting, and coordination at the laboratory–clinic interface as key requirements for scalable delivery (50). The pathway should also specify who reviews and communicates results, how negative, equivocal, and confirmed positive findings are distinguished, and who coordinates repeat sampling or imaging.

Standardized reporting and participation in EQA/PT would support consistency across centers (23, 27). In addition, established quality indicators for molecular tumor boards may provide a framework for multidisciplinary oversight, documentation, and accountability (57). These organizational safeguards should complement, rather than repeat, the patient-centered counselling and result-management measures described above.

Implementation should also be evaluated in terms of access and economic sustainability. Existing budget-impact and health-economic studies provide useful approaches for modeling assay costs, testing frequency, imaging triggers, and downstream care (48, 49). Cost-effectiveness analyses in advanced lung cancer offer additional examples of how liquid biopsy may influence diagnostic procedures, treatment selection, and overall healthcare expenditure (58, 59), but these findings cannot be directly extrapolated to MRD surveillance in EBC. Prospective implementation studies should therefore evaluate indication-specific costs, confirmatory testing, downstream resource use, patient-level burden, and access disparities alongside clinical and patient-reported outcomes.

Taken together, these operational lessons may inform the development of future MRD implementation pathways in EBC.

## 7 Future directions and conclusion

A top priority for the next phase is to validate, within prospectively designed trials and prespecified statistical frameworks, the relationship between ctDNA clearance and/or sustained ctDNA negativity and clinically meaningful outcomes such as iDFS and OS. Establishing this mapping is essential if ctDNA dynamics are to be evaluated as candidate intermediate or surrogate endpoints. Trial protocols and statistical analysis plans (SAPs) should therefore prespecify the target positivity rate, repeat-positivity rules, and rescue pathways. Cross-trial, patient-level analyses and methodological work from the ctMoniTR initiative have already proposed harmonized response definitions and sampling time points, and have shown across multiple tumor types that ctDNA clearance is associated with clinical benefit, offering a practical blueprint for early endpoint assessment and repeat-sampling strategies in prospective studies (60–62). However, these associations do not establish ctDNA clearance as a validated surrogate endpoint in EBC.

Transparent reporting, data and code availability, and participation in EQA/PT should remain priorities for improving the reproducibility and cross-center comparability of future MRD studies (14, 23, 27).

In the multi-omics and AI domain, a near-term priority is to evaluate complementary integration of methylation/fragmentomics with high-density variant tracking using WGS–powered approaches. The next step is to test, under harmonized endpoints and open code, whether integrated models—and machine-learning–based denoising or causal-inference methods—deliver incremental gains in clinical utility rather than improvements confined to analytical performance.

Future implementation studies should evaluate clinical feasibility and health-system sustainability together with patient acceptability and psychological burden (46, 47), as well as equitable access and patient-level costs (48–50). These outcomes should be assessed alongside analytical performance and clinical endpoints to determine whether longitudinal MRD surveillance can be implemented in a clinically useful, equitable, and acceptable manner.

In summary, five elements are likely to determine whether ctDNA-MRD moves beyond detection toward demonstrable patient benefit: prospective validation of MRD-guided intervention and candidate endpoints, transparent data and code, rigorous evaluation of integrated multi-omics and AI models, parallel advances in health economics and quality systems, and patient-centered implementation pathways that support informed decision-making and appropriate management of uncertain findings. Together, these priorities provide a practical agenda for future research and implementation in EBC.

## References

[1] Harbeck N Nitz UA Christgen M Kümmel S Braun M Schumacher C . De-escalated neoadjuvant trastuzumab-emtansine with or without endocrine therapy versus trastuzumab with endocrine therapy in HR+/HER2+ early breast cancer: 5-year survival in the WSG-ADAPT-TP trial. J Clin Oncol. (2023) 41:3796–804. doi: 10.1200/JCO.22.01816 36809046

[2] Early Breast Cancer Trialists’ Collaborative Group . Reductions in recurrence in women with early breast cancer entering clinical trials between 1990 and 2009: a pooled analysis of 155 746 women in 151 trials. Lancet. (2024) 404:1407–18. doi: 10.1016/S0140-6736(24)01745-8 39396348 PMC12979726

[3] Nader-Marta G Monteforte M Agostinetto E Cinquini M Martins-Branco D Langouo M . Circulating tumor DNA for predicting recurrence in patients with operable breast cancer: a systematic review and meta-analysis. ESMO Open. (2024) 9:102390. doi: 10.1016/j.esmoop.2024.102390 38460249 PMC10940943

[4] Lipsyc-Sharf M de Bruin EC Santos K McEwen R Stetson D Patel A . Circulating tumor DNA and late recurrence in high-risk hormone receptor-positive, human epidermal growth factor receptor 2-negative breast cancer. J Clin Oncol. (2022) 40:2408–19. doi: 10.1200/JCO.22.00908 35658506 PMC9467679

[5] Shaw JA Page K Wren E de Bruin EC Kalashnikova E Hastings R . Serial postoperative circulating tumor DNA assessment has strong prognostic value during long-term follow-up in patients with breast cancer. JCO Precis Oncol. (2024) 8:e2300456. doi: 10.1200/PO.23.00456 38691816 PMC11161241

[6] De Mattos-Arruda L Mayor R Ng CKY Weigelt B Martínez-Ricarte F Torrejon D . Cerebrospinal fluid-derived circulating tumour DNA better represents the genomic alterations of brain tumours than plasma. Nat Commun. (2015) 6:8839. doi: 10.1038/ncomms9839 26554728 PMC5426516

[7] Comino-Méndez I Velasco-Suelto J Pascual J López-López E Quirós-Ortega ME Gaona-Romero C . Identification of minimal residual disease using the clonesight test for ultrasensitive ctDNA detection to anticipate late relapse in early breast cancer. Breast Cancer Res. (2025) 27:65. doi: 10.1186/s13058-025-02016-7 40312346 PMC12044774

[8] Elliott MJ Kim J Dou A Fuentes Antrás J Amir E Nadler MB . Comprehensive tumor-agnostic evaluation of genomic and epigenomic-based approaches for the identification of circulating tumor DNA in early-stage breast cancer. ESMO Open. (2025) 10:105286. doi: 10.1016/j.esmoop.2025.105286 40472659 PMC12172975

[9] Garcia-Murillas I Abbott CW Cutts RJ Boyle SM Pugh J Keough KC . Whole genome sequencing-powered ctDNA sequencing for breast cancer detection. Ann Oncol. (2025) 36:673–81. doi: 10.1016/j.annonc.2025.01.021 39914664

[10] Turner N Pimentel I Cescon D Iwata H Janni W Lacko A . Abstract GS3-01: Circulating tumor DNA surveillance in ZEST, a randomized, phase 3, double-blind study of niraparib or placebo in patients w/ triple-negative breast cancer or HER2+ BRCA-mutated breast cancer with molecular residual disease after definitive therapy. Clin Cancer Res. (2025) 31:GS3-01. doi: 10.1158/1557-3265.SABCS24-GS3-01 36230740

[11] American Association for Cancer Research . ZEST Trial Offers Insights for Using Ctdna to Predict Breast Cancer Recurrence (2024). Available online at: https://www.aacr.org/about-the-aacr/newsroom/news-releases/zest-trial-offers-insights-for-using-ctdna-to-predict-breast-cancer-recurrence/ (Accessed August 1, 2025).

[12] Turner NC Swift C Jenkins B Kilburn L Coakley M Beaney M . Results of the c-TRAK TN trial: a clinical trial utilising ctDNA mutation tracking to detect molecular residual disease and trigger intervention in patients with moderate- and high-risk early-stage triple-negative breast cancer. Ann Oncol. (2023) 34:200–11. doi: 10.1016/j.annonc.2022.11.005 36423745

[13] Abbosh C Swanton C Birkbak NJ . Clonal haematopoiesis: a source of biological noise in cell-free DNA analyses. Ann Oncol. (2019) 30:358–9. doi: 10.1093/annonc/mdy552 30649226 PMC6442654

[14] Panet F Papakonstantinou A Borrell M Vivancos J Vivancos A Oliveira M . Use of ctDNA in early breast cancer: analytical validity and clinical potential. NPJ Breast Cancer. (2024) 10:50. doi: 10.1038/s41523-024-00653-3 38898045 PMC11187121

[15] Shen SY Singhania R Fehringer G Chakravarthy A Roehrl MHA Chadwick D . Sensitive tumour detection and classification using plasma cell-free DNA methylomes. Nature. (2018) 563:579–83. doi: 10.1038/s41586-018-0703-0 30429608

[16] Cristiano S Leal A Phallen J Fiksel J Adleff V Bruhm DC . Genome-wide cell-free DNA fragmentation in patients with cancer. Nature. (2019) 570:385–9. doi: 10.1038/s41586-019-1272-6 31142840 PMC6774252

[17] Mouliere F Chandrananda D Piskorz AM Moore EK Morris J Ahlborn LB . Enhanced detection of circulating tumor DNA by fragment size analysis. Sci Transl Med. (2018) 10:eaat4921. doi: 10.1126/scitranslmed.aat4921 30404863 PMC6483061

[18] Snyder MW Kircher M Hill AJ Daza RM Shendure J . Cell-free DNA comprises an *in vivo* nucleosome footprint that informs its tissues-of-origin. Cell. (2016) 164:57–68. doi: 10.1016/j.cell.2015.11.050 26771485 PMC4715266

[19] Sun K Jiang P Wong AIC Cheng YKY Cheng SH Zhang H . Size-tagged preferred ends in maternal plasma DNA shed light on the production mechanism and show utility in noninvasive prenatal testing. Proc Natl Acad Sci. (2018) 115:E5106–14. doi: 10.1073/pnas.1804134115 29760053 PMC5984542

[20] Newman AM Lovejoy AF Klass DM Kurtz DM Chabon JJ Scherer F . Integrated digital error suppression for improved detection of circulating tumor DNA. Nat Biotechnol. (2016) 34:547–55. doi: 10.1038/nbt.3520 27018799 PMC4907374

[21] Janni W Rack B Friedl TWP Hartkopf AD Wiesmüller L Pfister K . Detection of minimal residual disease and prediction of recurrence in breast cancer using a plasma-only circulating tumor DNA assay. ESMO Open. (2025) 10:104296. doi: 10.1016/j.esmoop.2025.104296 40120523 PMC11982450

[22] Bartolomucci A Nobrega M Ferrier T Dickinson K Kaorey N Nadeau A . Circulating tumor DNA to monitor treatment response in solid tumors and advance precision oncology. NPJ Precis Oncol. (2025) 9:84. doi: 10.1038/s41698-025-00876-y 40122951 PMC11930993

[23] De Jager VD Giacomini P Fairley JA Toledo RA Patton SJ Joosse SA . Reporting of molecular test results from cell-free DNA analyses: expert consensus recommendations from the 2023 European Liquid Biopsy Society ctDNA Workshop. eBioMedicine. (2025) 114:105636. doi: 10.1016/j.ebiom.2025.105636 40121940 PMC11979934

[24] Schmitt MW Kennedy SR Salk JJ Fox EJ Hiatt JB Loeb LA . Detection of ultra-rare mutations by next-generation sequencing. Proc Natl Acad Sci. (2012) 109:14508–13. doi: 10.1073/pnas.1208715109 22853953 PMC3437896

[25] Adalsteinsson VA Ha G Freeman SS Choudhury AD Stover DG Parsons HA . Scalable whole-exome sequencing of cell-free DNA reveals high concordance with metastatic tumors. Nat Commun. (2017) 8:1324. doi: 10.1038/s41467-017-00965-y 29109393 PMC5673918

[26] Chen X Chang C-W Spoerke JM Yoh KE Kapoor V Baudo C . Low-pass whole-genome sequencing of circulating cell-free DNA demonstrates dynamic changes in genomic copy number in a squamous lung cancer clinical cohort. Clin Cancer Res. (2019) 25:2254–63. doi: 10.1158/1078-0432.CCR-18-1593 30617129

[27] Van Der Leest P Rozendal P Hinrichs J Van Noesel CJM Zwaenepoel K Deiman B . External quality assessment on molecular tumor profiling with circulating tumor DNA-based methodologies routinely used in clinical pathology within the COIN Consortium. Clin Chem. (2024) 70:759–67. doi: 10.1093/clinchem/hvae014 38484302

[28] Garcia-Murillas I Cutts RJ Walsh-Crestani G Phillips E Hrebien S Dunne K . Longitudinal monitoring of circulating tumor DNA to detect relapse early and predict outcome in early breast cancer. Breast Cancer Res Treat. (2025) 209:493–502. doi: 10.1007/s10549-024-07508-2 39424680 PMC11785695

[29] Zaikova E Cheng BYC Cerda V Kong E Lai D Lum A . Circulating tumour mutation detection in triple-negative breast cancer as an adjunct to tissue response assessment. NPJ Breast Cancer. (2024) 10:3. doi: 10.1038/s41523-023-00607-1 38182588 PMC10770342

[30] Verschoor N Bos MK Oomen-de Hoop E Martens JWM Sleijfer S Jager A . A review of trials investigating ctDNA-guided adjuvant treatment of solid tumors: the importance of trial design. Eur J Cancer. (2024) 207:114159. doi: 10.1016/j.ejca.2024.114159 38878446

[31] Alba-Bernal A Godoy-Ortiz A Domínguez-Recio ME López-López E Quirós-Ortega ME Sánchez-Martín V . Increased blood draws for ultrasensitive ctDNA and CTCs detection in early breast cancer patients. NPJ Breast Cancer. (2024) 10:36. doi: 10.1038/s41523-024-00642-6 38750090 PMC11096188

[32] Pontolillo L Gouda MA Venetis K Nicolò E Reduzzi C . Cancer in a drop: liquid biopsy highlights from San Antonio Breast Cancer Symposium (SABCS) 2024. J Liq Biopsy. (2025) 7:100286. doi: 10.1016/j.jlb.2025.100286 40027228 PMC11863971

[33] Berger F Marce M Delaloge S Hardy-Bessard A-C Bachelot T Bièche I . Randomised, open-label, multicentric phase III trial to evaluate the safety and efficacy of palbociclib in combination with endocrine therapy, guided by ESR1 mutation monitoring in oestrogen receptor-positive, HER2-negative metastatic breast cancer patients: study design of PADA-1. BMJ Open. (2022) 12:e055821. doi: 10.1136/bmjopen-2021-055821 35241469 PMC8896060

[34] Bidard F-C Hardy-Bessard A-C Dalenc F Bachelot T Pierga J-Y De La Motte Rouge T . Switch to fulvestrant and palbociclib versus no switch in advanced breast cancer with rising ESR1 mutation during aromatase inhibitor and palbociclib therapy (PADA-1): a randomised, open-label, multicentre, phase 3 trial. Lancet Oncol. (2022) 23:1367–77. doi: 10.1016/S1470-2045(22)00555-1 36183733

[35] Parsons HA Blewett T Chu X Sridhar S Santos K Xiong K . Circulating tumor DNA association with residual cancer burden after neoadjuvant chemotherapy in triple-negative breast cancer in TBCRC 030. Ann Oncol. (2023) 34:899–906. doi: 10.1016/j.annonc.2023.08.004 37597579 PMC10898256

[36] Mittendorf EA Assaf ZJ Harbeck N Zhang H Saji S Jung KH . Peri-operative atezolizumab in early-stage triple-negative breast cancer: final results and ctDNA analyses from the randomized phase 3 IMpassion031 trial. Nat Med. (2025) 31:2397–404. doi: 10.1038/s41591-025-03725-4 40467898 PMC12283416

[37] Lu Y Ren L Yang M Liu J . Clinical management of circulating tumor DNA in breast cancer: detection, prediction, and monitoring. Breast Cancer: Targets Ther. (2025) 17:851–61. doi: 10.2147/BCTT.S542704 41036092 PMC12479222

[38] Coakley M Villacampa G Sritharan P Swift C Dunne K Kilburn L . Comparison of circulating tumor DNA assays for molecular residual disease detection in early-stage triple-negative breast cancer. Clin Cancer Res. (2024) 30:895–903. doi: 10.1158/1078-0432.CCR-23-2326 38078899 PMC10870111

[39] Cutts R Ulrich L Beaney M Robert M Coakley M Bunce C . Association of post-operative ctDNA detection with outcomes of patients with early breast cancers. ESMO Open. (2024) 9:103687. doi: 10.1016/j.esmoop.2024.103687 39216186 PMC11402396

[40] Tie J Wang Y Lo SN Lahouel K Cohen JD Wong R . Circulating tumor DNA analysis guiding adjuvant therapy in stage II colon cancer: 5-year outcomes of the randomized DYNAMIC trial. Nat Med. (2025) 31:1509–18. doi: 10.1038/s41591-025-03579-w 40055522 PMC12974608

[41] Tie J Cohen JD Lahouel K Lo SN Wang Y Kosmider S . Circulating tumor DNA analysis guiding adjuvant therapy in stage II colon cancer. N Engl J Med. (2022) 386:2261–72. doi: 10.1056/NEJMoa2200075 35657320 PMC9701133

[42] U.S. Food and Drug Administration . Use of Circulating Tumor Deoxyribonucleic Acid for Early-Stage Solid Tumor Drug Development: Guidance for Industry (2024). Available online at: https://www.fda.gov/regulatory-information/search-fda-guidance-documents/use-circulating-tumor-deoxyribonucleic-acid-early-stage-solid-tumor-drug-development-guidance (Accessed August 23, 2025).

[43] ClinicalTrials.gov . Ctdna-Guided De-Escalation of Adjuvant Chemotherapy With Dalpiciclib in HR-Positive/HER2-Negative Breast Cancer. Clinical Trial Registration NCT06970912. Available online at: https://clinicaltrials.gov/study/NCT06970912 (Accessed August 23, 2025).

[44] ClinicalTrials.gov . A Single Arm Phase II Trial of Circulating Tumor DNA-Guided Adjuvant Therapy With Elacestrant in Adults With Hormone Receptor-Positive, HER2-Negative Breast Cancers at Risk for Late Recurrence (CATE). Clinical Trial Registration NCT06923527. Available online at: https://clinicaltrials.gov/study/NCT06923527 (Accessed August 23, 2025).

[45] Friends of Cancer Research . Framework for Integrating Change in Ctdna Levels in Advanced Cancer Clinical Trials to Support Meta-Analyses for Intermediate Endpoint Validation (2024). Available online at: https://friendsofcancerresearch.org/wp-content/uploads/Framework-for-Integrating-Change-in-ctDNA-Levels-in-Advanced-Cancer-Clinical-Trials-to-Support-Meta-analyses-for-Intermediate-Endpoint-Validation.pdf (Accessed October 11, 2025).

[46] Woof VG Lee RJ Lorigan P French DP . Circulating tumour DNA monitoring and early treatment for relapse: views from patients with early-stage melanoma. Br J Cancer. (2022) 126:1450–6. doi: 10.1038/s41416-022-01766-x 35301436 PMC8927744

[47] Adi-Wauran E Clausen M Shickh S Gagliardi AR Denburg A Oldfield LE . I just wanted more”: hereditary cancer syndromes patients’ perspectives on the utility of circulating tumour DNA testing for cancer screening. Eur J Hum Genet. (2024) 32:176–81. doi: 10.1038/s41431-023-01473-y 37821757 PMC10853540

[48] Li Y Heer AK Sloane HS Edelstein DL Tie J Gibbs P . Budget impact analysis of circulating tumor DNA testing for colon cancer in commercial health and Medicare Advantage plans. JAMA Health Forum. (2024) 5:e241270. doi: 10.1001/jamahealthforum.2024.1270 38819797 PMC11143467

[49] Rui M Wang Y You JHS . Health economic evaluations of circulating tumor DNA testing for cancer screening: systematic review. Cancer Med. (2025) 14:e70641. doi: 10.1002/cam4.70641 39907177 PMC11795416

[50] Sheriff S Saba M Patel R Fisher G Schroeder T Arnolda G . A scoping review of factors influencing the implementation of liquid biopsy for cancer care. J Exp Clin Canc Res. (2025) 44:50. doi: 10.1186/s13046-025-03322-w 39934875 PMC11817833

[51] Bardia A Cortés J Bidard F-C Neven P Garcia-Sáenz J Aftimos P . Elacestrant in ER+, HER2- metastatic breast cancer with ESR1-mutated tumors: Subgroup analyses from the phase III EMERALD trial by prior duration of endocrine therapy plus CDK4/6 inhibitor and in clinical subgroups. Clin Cancer Res. (2024) 30:4299–309. doi: 10.1158/1078-0432.CCR-24-1073 39087959 PMC11443208

[52] Bidard F-C Kaklamani VG Neven P Streich G Montero AJ Forget F . Elacestrant (oral selective estrogen receptor degrader) versus standard endocrine therapy for estrogen receptor-positive, human epidermal growth factor receptor 2-negative advanced breast cancer: Results from the randomized phase III EMERALD trial. J Clin Oncol. (2022) 40:3246–56. doi: 10.1200/JCO.22.00338 35584336 PMC9553388

[53] Valenza C Trapani D Bidard F-C Gligorov J Cortés J Turner N . Elacestrant in ESR1-mutant, endocrine-responsive metastatic breast cancer: Should health authorities consider post hoc data to inform priority access? ESMO Open. (2024) 9:103701. doi: 10.1016/j.esmoop.2024.103701 39232441 PMC11403265

[54] East Genomics . Interim Esr1 Genomic Sample Routing Pathway (2025). Available online at: https://www.eastgenomics.nhs.uk/for-healthcare-professionals/updates/interim-esr1-routing/ (Accessed October 13, 2025).

[55] North Thames Genomic Medicine Service . ESR1 Ctdna Testing for Breast Cancer (2025). Available online at: https://norththamesgenomics.nhs.uk/esr1-ctdna-testing-for-breast-cancer/ (Accessed October 13, 2025).

[56] NHS England . Implementation of Genomic Testing for ESR1 Variants: Interim ESR1 Genomic Sample Routing Pathway, 20 January 2025–31 March 2025 (2025). Available online at: https://ney-genomics.org.uk/wp-content/uploads/2025/01/ESR1-testing-communication-for-providers-17.01.25-.pdf (Accessed October 13, 2025).

[57] Westphalen CB Boscolo Bielo L Aftimos P Beltran H Benary M Chakravarty D . ESMO Precision Oncology Working Group recommendations on the structure and quality indicators for molecular tumour boards in clinical practice. Ann Oncol. (2025) 36:614–25. doi: 10.1016/j.annonc.2025.02.009 40194904

[58] Isla D Álvarez R Arnal M Arriola E Azkarate A Azkona E . Detection of genomic alterations in liquid biopsies from patients with non-small cell lung cancer using FoundationOne Liquid CDx: A cost-effectiveness analysis. J Med Econ. (2024) 27:1379–87. doi: 10.1080/13696998.2024.2413289 39387325

[59] Liu S Graves N Tan AC . The cost-effectiveness of including liquid biopsy into molecular profiling strategies for newly diagnosed advanced non-squamous non-small cell lung cancer in an Asian population. Lung Cancer. (2024) 191:107794. doi: 10.1016/j.lungcan.2024.107794 38636314

[60] Andrews HS Zariffa N Nishimura KK Deng Y Eisele M Ensor J . Molecular response cutoffs and ctDNA collection timepoints influence on interpretation of associations between early changes in ctDNA and overall survival in patients treated with anti-PD(L)1 and/or chemotherapy. J Immunother Cancer. (2025) 13:e012454. doi: 10.1136/jitc-2025-012454 40983343 PMC12458711

[61] Andrews HS Zariffa N Nishimura KK Choi SH Deng S Eisele M . ctDNA clearance as an early indicator of improved clinical outcomes in advanced NSCLC treated with TKI: Findings from an aggregate analysis of eight clinical trials. Clin Cancer Res. (2025) 31:2162–72. doi: 10.1158/1078-0432.CCR-24-3612 40261185 PMC12130803

[62] Niu S Sun T Wang M Yao L He T Wang Y . Multiple time points for detecting circulating tumor DNA to monitor the response to neoadjuvant therapy in breast cancer: A meta-analysis. BMC Cancer. (2025) 25:115. doi: 10.1186/s12885-025-13526-0 39844103 PMC11752932

