# Supplementary material for: From detection to action: ctDNA-MRD surveillance and translational strategies in early breast cancer
Source: Front Oncol. 2026 Aug 24;16:1850732. doi: 10.3389/fonc.2026.1850732 (PMC13548034; doi:10.3389/fonc.2026.1850732)
Supplement: Supplementary file 1 [file Table1.docx]

Supplementary Material

# 1 Supplementary Tables

| **Symbol** | **Description** | **Example value** | **Source/Comment** |
| --- | --- | --- | --- |
| Δ (months) | Sampling interval between ctDNA draws (e.g. 3 or 6 months). | 3 or 6 | Design choice for surveillance schedule. |
| N | Cohort size of high-risk early breast cancer patients under follow-up. | 100 | Placeholder; adapt to trial or real-world population. |
| p_MRD | Annual probability of true molecular relapse (MRD) in the target cohort. | 0.15 | Can be informed by recent ctDNA meta-analyses in early breast cancer. |
| S | Assay sensitivity to detect MRD within the biological lead-time window when sampled. | 0.9 | Based on performance of contemporary ctDNA-MRD platforms. |
| PPV | Positive predictive value of a repeat ctDNA-positive result for distant recurrence. | 1 | Short-term PPV can approach 100% in LIBERATE-like settings. |
| L (months) | Average biological lead time from first ctDNA positivity to clinical/radiologic relapse. | 5 | Can be approximated from longitudinal MRD studies by subtype. |
| Delay(3) | Expected sampling delay from relapse onset to first positive with a q3-month schedule. | 1.5 | Approximated as Δ/2 when L ≫ Δ. |
| Delay(6) | Expected sampling delay from relapse onset to first positive with a q6-month schedule. | 3 | Approximated as Δ/2 when L ≫ Δ. |
| C_test | Unit cost per ctDNA test (including blood draw and laboratory processing). | context-specific | Insert local tariff or trial cost. |
| C_img | Unit cost per indicated imaging assessment triggered by ctDNA. | context-specific | Insert cost of the imaging bundle used for confirmation. |
| ρ_img | Proportion of repeat-positive patients who proceed to indicated imaging. | 1 | Often assumed to be 1.0 in base-case analyses. |
| ε | Small constant to avoid division by zero in ICER calculations. | 0.001 | Technical parameter; does not affect results when ΔBenefit > 0. |

## Supplementary Table 1. Input parameters for the simplified q3 vs q6 ctDNA-MRD monitoring model.

| **Outcome** | **Equation** | **Interpretation / notes** |
| --- | --- | --- |
| Tests(Δ) | Tests(Δ) = 12 / Δ | Number of ctDNA blood draws per person-year under interval Δ (in months). |
| Capture(Δ) | Capture(Δ) ≈ S × [1 − Delay(Δ) / L] | Approximate probability that a true MRD event is sampled before clinical/radiologic relapse; approximation holds when L ≫ Δ. |
| Pos(Δ) | Pos(Δ) = N × p_MRD × Capture(Δ) × PPV | Expected number of patients per year with repeat ctDNA-positive MRD eligible for pre-emptive intervention. |
| Imgs(Δ) | Imgs(Δ) = Pos(Δ) × ρ_img | Number of imaging assessments triggered by ctDNA per year. |
| Cost(Δ) | Cost(Δ) = N × Tests(Δ) × C_test + Imgs(Δ) × C_img | Annual programme cost for ctDNA-guided surveillance and triggered imaging. |
| ΔBenefit | ΔBenefit = Pos(3) − Pos(6) | Incremental number of actionable MRD-positive patients detected with q3 versus q6 monitoring. |
| ICER | ICER = [Cost(3) − Cost(6)] / max{ΔBenefit, ε} | Incremental cost per additional actionable MRD-positive patient when switching from q6 to q3 monitoring. |
| ICER_LT (optional) | ICER_LT = [Cost(3) − Cost(6)] / [ΣLT_q3 − ΣLT_q6] | Incremental cost per additional month of total lead time gained, if cumulative lead-time data are available. |

## **Supplementary Table 2. Equations and outputs for the simplified q3 vs q6 ctDNA-MRD monitoring model.**
